# Rhizosphere Soil Bacterial Communities of Continuous Cropping-Tolerant and Sensitive Soybean Genotypes Respond Differently to Long-Term Continuous Cropping in Mollisols

**DOI:** 10.3389/fmicb.2021.729047

**Published:** 2021-09-13

**Authors:** Ming Yuan, Taobing Yu, Qihan Shi, Dongwei Han, Kanchao Yu, Lianxia Wang, Shurong Wang, Hao Xiang, Ronghui Wen, Hai Nian, Tengxiang Lian

**Affiliations:** ^1^Qiqihar Branch of Heilongjiang Academy of Agricultural Sciences, Qiqihar, China; ^2^The State Key Laboratory for Conservation and Utilization of Subtropical Agro-bioresources, South China Agricultural University, Guangzhou, China; ^3^The Key Laboratory of Plant Molecular Breeding of Guangdong Province, College of Agriculture, South China Agricultural University, Guangzhou, China; ^4^Institute of Hydrobiology, Chinese Academy of Sciences, Wuhan, China; ^5^The State Key Laboratory for Conservation and Utilization of Subtropical Agro-bioresources, College of Life Science and Technology, Guangxi University, Nanning, China

**Keywords:** continuous cropping, soybean, Mollisol, rhizosphere microorganisms, network

## Abstract

The continuous planting of soybeans leads to soil acidification, aggravation of soil-borne diseases, reduction in soil enzyme activity, and accumulation of toxins in the soil. Microorganisms in the rhizosphere play a very important role in maintaining the sustainability of the soil ecosystem and plant health. In this study, two soybean genotypes, one bred for continuous cropping and the other not, were grown in a Mollisol in northeast China under continuous cropping for 7 and 36years in comparison with soybean–maize rotation, and microbial communities in the rhizosphere composition were assessed using high-throughput sequencing technology. The results showed that short- or long-term continuous cropping had no significant effect on the rhizosphere soil bacterial alpha diversity. Short-term continuous planting increased the number of soybean cyst nematode (*Heterodera glycines*), while long-term continuous planting reduced these numbers. There were less soybean cyst nematodes in the rhizosphere of the tolerant genotypes than sensitive genotypes. In addition, continuous cropping significantly increased the potential beneficial bacterial populations, such as *Pseudoxanthomonas*, *Nitrospira*, and *Streptomyces* compared to rotation and short-term continuous cropping, suggesting that long-term continuous cropping of soybean shifts the microbial community toward a healthy crop rotation system. Soybean genotypes that are tolerant to soybean might recruit some microorganisms that enhance the resistance of soybeans to long-term continuous cropping. Moreover, the network of the two genotypes responded differently to continuous cropping. The tolerant genotype responded positively to continuous cropping, while for the sensitive genotype, topology analyses on the instability of microbial community in the rhizosphere suggested that short periods of continuous planting can have a detrimental effect on microbial community stability, although this effect could be alleviated with increasing periods of continuous planting.

## Introduction

As one of the most important soil resources in China, Mollisols in the northeast play a crucial role in maintaining domestic food demand. Soybean [*Glycine max (L.)* Merrill] is one of the most important food crops in the world, providing a large amount of oil and protein for humans and animals (Chigen et al., 2018; Liu et al., 2020). There is a large demand for soybeans, especially in China (Liu et al., 2020). Due to the limited arable land, climatic conditions, and large proportion of arable land with other crops, soybeans are often continuously planted in this region (Liu et al., 2012). On some farms, soybeans have been planted continuously for 40years or even longer (Liu et al., 2020). The continuous planting of soybeans leads to soil acidification, aggravation of soil-borne diseases, reduction in soil enzyme activity, and accumulation of toxins in the soil (Zhan et al., 2004; Yan et al., 2012; Pérez-Brandán et al., 2014; Bai et al., 2015). Several studies have clarified that these changes are significantly related to biological factors in the soil (Ji et al., 1996; Dias et al., 2015; Liu et al., 2020).

Rhizosphere microorganisms play a very important role in maintaining the sustainability of the soil ecosystem and plant health (Waldrop et al., 2000; Avidano et al., 2005). Different cropping systems can significantly change the microbial community structure (Meriles et al., 2009; Zhou et al., 2018; Liu et al., 2019). However, these changes depend on the type of cropping system, soil type, and crop species. For instance, both Tang et al. (2009) and Zhu et al. (2014) found that, compared with crop rotation systems, the abundance of Actinobacteria significantly decreased under the continuous cropping of soybean. Xu et al. (1995) found that continuous cropping decreased the abundance of *Penicillium* sp., while it increased the abundance of *Fusarium* sp. However, another study showed that, compared with crop rotation, continuous soybean cropping did not change the microbial community structure (Hu and Wang, 1996). The inconsistencies between these studies are mainly due to the differences in soil types, research methods, crop rotation systems, and years of continuous cropping. At the same time, the mechanisms associated with continuous cropping obstacles are complex and need to be explored in greater depth under different conditions.

Soybean is one of the most sensitive crops to continuous cropping. Studies have shown that continuous cropping of soybean for 3years could reduce yield by approximately 30% (Liu et al., 2020). In addition, compared to crop rotation, soybean root rot diseases and cyst nematodes in soybean fields increased significantly in a short-term continuously cropped system (Cai et al., 2015). However, root rot and cystic nematode disease in soybean fields might be weakened after long-term continuous cropping (Song et al., 2017). This might be attributed to the fact that long-term continuous cropping could increase the population of beneficial microorganisms, which could help inhibit the colonization and development of pathogens in the soil (Wei et al., 2015; Liu et al., 2020). For example, Liu et al. (2020) found that 13years of long-term continuously cropped soybean increased beneficial bacteria, such as *Bradyrhizobium* sp., *Gemmatimonas* sp., *Mortierella* sp., and *Paecilomyces* sp. and decreased the pathogenic fungi of *Fusarium* sp. However, some researchers also found that long-term continuous of soybeans may reduce the populations of beneficial microorganisms such as *Trichoderma*, *Colloids*, and *Pseudomonas fluorescens* (Pérez-Brandán et al., 2014). Therefore, the change in the microbial community caused by continuous or rotational cropping is crucial to reveal the mechanisms underlying obstacles to continuous soybean cropping. In addition, the inconsistency of these studies indicates that more research on the soil microbial community needs to be performed.

The rhizosphere microbial community of crops is also affected by genotypes (Kaisermann et al., 2017; Lian et al., 2019). Resistant genotypes can recruit some beneficial microorganisms to help the host resist various stresses (Lebeis et al., 2015; Kwak et al., 2018). For example, tomato genotypes that are resistant to *Fusarium* wilt can recruit a large number of *Flavobacterium* to help alleviate the symptoms of *Fusarium* wilt (Kwak et al., 2018). Our previous studies have also shown that aluminium-resistant soybeans can recruit *Tumebacillus* and *Burkholderia* and alleviate aluminium toxicity (Lian et al., 2019). Regarding the continuous cropping of soybean, although microorganisms are known to play a very important role in alleviating or aggravating continuous cropping obstacles, the comparison of the microbial community in the rhizosphere among different genotypes has not been reported. Understanding the characteristics of the rhizosphere microbial community of continuous-resistant genotype soybeans can help us understanding how those resistant genotypes could better adapt to continuous cropping.

Here, we aimed to characterize the rhizosphere microorganisms of different soybean genotypes, one bred for continuous cropping and one not, grown in a Mollisol in northeast China under continuous cropping for 7 and 36years with soybean–maize rotation. Based on the higher adaptability of continuous cropping-tolerant (CC-T) soybean genotypes to continuous cropping, we hypothesized that (1) the bacterial diversity in the rhizosphere of CC-T genotypes would be higher than that of continuous cropping-sensitive (CC-S) genotypes and that (2) the response of bacterial community structure to continuous cropping between CC-T and CC-S soybean genotypes would be different. CC-T may recruit beneficial microbial species to alleviate the occurrence of soil-borne diseases.

## Materials and Methods

### Soil Source and Plant Materials

To assess the responses of continuous cropping-tolerant and cropping-sensitive soybean genotypes soil used in this study was a Mollisol, according to USDA soil taxonomy. Soybeans used in the experiment included Qinong1 and Qinong5, which were continuous cropping-tolerant (CCT), and Heihe43 and Henong76, which were continuous cropping-sensitive (CCS).

### Experimental Design and Plant and Rhizosphere Soil Collection

Continuous cropping of two soybean genotypes for 7 and 36years, as well as treatment of soybean-corn rotations for 10years, were selected for this study. The total area of the experimental field (47°15′N, 123°40′E) was 2ha, including 1.3ha of 36-year continuous cropping and 0.3ha each of 7-year continuous and rotation cropping (Supplementary Figure S1). A total of 72 rhizosphere soil samples (six treatments×12 replicates) were collected at the flowering stage of soybean on July 15, 2020. All rhizosphere soil samples were collected by gently shaking the plant root to remove loosely attached soil, and then the soil adhering to the root system was transported to an aseptic bag. Two grams of rhizosphere soil from each sample was placed in a sterilized microcentrifuge tube and stored at −80°C for DNA extraction. The shoots and roots were separated for biomass measurements. All the soybean cyst nematodes (*Heterodera glycines*) and nodules from the roots were counted after removal from the root system. In brief, after the soybean cyst nematode growth broke through the root surface of soybean (35days after seedling), 10 soybean plants were randomly selected for the soybean cyst nematodes counting. The yield per square meter was measured after soybean maturity.

### Soil DNA Extraction and Next-Generation Sequencing

In total, 72 rhizosphere soil samples were used for sequencing. Soil total DNA was extracted using the Fast DNA SPIN Kit for Soil (MP Biomedicals, Santa Ana, CA). Primer sequences of 341F/805R were used to amplify the V4 (V3–V4) hypervariable regions of the 16S rRNA. The PCR products were purified and then sequenced using the MiSeq Illumina platform (Illumina, United States) at Icongene (Wuhan) Gene Technology Co., Ltd. (Wuhan, China). All sequences were deposited into the GenBank short-read archive PRJNA732989.

After sequencing, the raw FASTQ files were processed using QIIME Pipeline Version 1.19.1. Briefly, all sequence reads were assigned to each sample based on the barcodes. Sequences with low quality (length<200bp and average base quality score<20) were removed before further analysis. The chimera of trimmed sequences was detected and removed using the UCHIME algorithm (Edgar et al., 2011). The sequences were phylogenetically assigned according to their best matches to sequences in the RDP database using the RDP classifier (Cole et al., 2009). Operational taxonomic units (OTUs) were classified at 97% sequence similarity using CD-HIT (Li and Godzik, 2015). Chao1 richness and Shannon’s diversity index were calculated in QIIME. Constrained principal coordinate analysis (CPCoA) and significance tests (Adonis test and mantel test) were performed in program R version 3.5.1 for Windows with the “vegan” package. Ternary plots were constructed to show the abundance comparison of OTUs (>5%) for rotation system, and continuous cropping for 7 and 36years of the two genotypes soybeans, using the “vcd” package (Friendly and Meyer, 2015). One-way analysis of variance (ANOVA) was used in GenStat 13 (VSN International, Hemel Hempstead, United Kingdom) to assess the relative abundances of different taxonomic levels of bacteria. Additionally, a Pearson bivariate correlation analysis was performed to access the correlations between pH, soybean cyst nematode, yield, and the genera with higher relative abundance.

### Core Bacteria and Co-occurrence Network Analyses

Core bacteria, which contain a list of OTUs observed in 60% of all rhizosphere samples, were obtained by MicrobiomeAnalyst (Chong et al., 2020). Bacterial co-occurrence networks were analyzed for each cropping system. To study the network structure of OTUs with high abundance, we selected OTUs with more than 0.2% relative abundance to calculate Spearman’s rank correlation coefficients. The correlations between OTUs were selected at *p*<0.05 and Spearman’s correlation coefficient of more than 0.8 (Mendes et al., 2018). The nodes and edges represent bacterial OTUs and the correlations between bacterial OTUs, respectively. Statistical analyses were calculated using the “psych” package in R and then visualized in Gephi (Jiang et al., 2017). Keystone species were defined according to high node degree, high betweenness centrality, and high closeness centrality (Berry and Widder, 2014; Agler et al., 2016). The NetShift analysis was performed using the NetShift Software tool^1^ to compare the networks of different treatments and find the “driver microbes” between the treatment of soybean-corn rotations and 7, 36years continuous cropping for the sensitive and tolerant genotypes, respectively (Kuntal et al., 2019).

## Results

### Soybean Cyst Nematode Numbers and Yield

Both cropping systems and soybean genotypes significantly affected the soybean cyst nematode number and yield (Table 1). There was a higher cyst nematode number in the sensitive genotypes than in the tolerant genotypes. In particular, for the sensitive genotypes, the soybean cyst nematode number significantly increased in the 7-year continuous cropping field but markedly decreased in the 36-year continuous cropping field. However, continuous cropping had no significant effect on the tolerant genotypes. In addition, the yield increased in the 7-year continuous cropping field but decreased in the 36-year continuous cropping field, with a higher yield for the tolerant genotypes than the sensitive genotypes (Table 1).

**Table 1:** Soil pH, number of cyst nematode, shoot and root biomass, and yield of different treatments.

	CKT	CKS	7T	7S	36T	36S	Cropping systems	Genotype	Cropping systems*Genotype	LSD
pH	7.08±0.08b	6.91±0.1c	6.91±0.04c	7.29±0.13a	6.69±0.12d	6.66±0.07d	<0.001^***^	0.075	<0.001^***^	0.005
Number of cyst nematode	1.5±1.64bc	5.5±1.64b	1.67±1.86bc	15.17±8.84a	0.33±0.52c	4.67±1.63bc	0.002^**^	<0.001^***^	0.007^**^	2.236
Shoot biomass	88.27±32.24ab	59.29±9.9b	81.63±25.51b	79.13±16.13b	115.89±23.18a	88.33±24.65b	0.014^*^	0.016^*^	0.301	27.2
Root biomass (g)	10.7±4.29a	10.6±2.29a	11.5±4.35a	10.41±2.33a	11.85±2.99a	11.82±3.85a	0.686	0.72	0.91	1.99
Yield (g/m^2^)	353±35.34cd	293±17.38d	481±45.95a	401±46.82bc	456±35.3ab	351±27.91cd	<0.001^***^	<0.001^***^	0.683	74.1

CKT: crop rotation-tolerance, CKS: crop rotation-sensitive, 7T: 7year-continuous cropping-tolerant, 7S: 7year-continuous cropping-sensitive, 36T: 36year-continuous cropping-tolerant, 36S: 36year-continuous cropping-sensitive.

* *p*<0.05;

** *p*<0.01;

*** *p*<0.001.

### Rhizosphere Soil Bacterial Diversity

**Figure 1:**
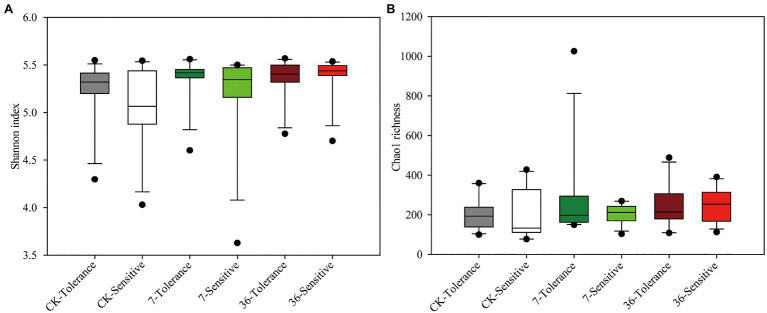
Rhizobacterial community Shannon index **(A)** and Chao1 richness **(B)** of different treatments. One-way ANOVA, *n*=12, *p*<0.05.

**Figure 2:**
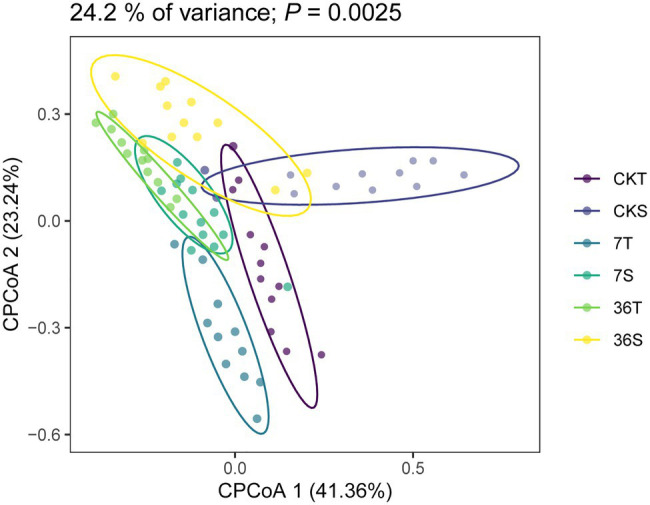
Constrained Principal coordinate analysis (CPCoA) based on Bray-Curtis dissimilarities of 16S rRNA diversity in the rhizosphere.

Both cropping systems and soybean genotypes had no significant effect on the alpha diversity of rhizobacterial communities (one-way ANOVA, *p*>0.05; Figures 1A,B). Regarding beta diversity, CPCoA and two-way PERMANOVA revealed that cropping systems and soybean genotypes interactively affected the rhizobacterial communities (PERMANOVA, *p*<0.05; Figure 2; Supplementary Figure S2; Table 2). In more detail, rhizobacterial community structures differed between tolerant and sensitive soybean genotypes irrespective of cropping systems (Supplementary Figure S3; Table 2).

**Table 2:** Effects of cropping systems and soybean genotypes on the differentiation of bacterial communities based on PERMANOVA.

Factor	F	*R* ^2^	*p*
Cropping systems	4.3253	0.05819	0.001^***^
Genotype	2.3132	0.03199	0.024^*^
Cropping systems: Genotype	4.2608	0.05738	0.002^**^
36T vs. 36S	1.615	0.06839	0.058
7T vs. 7S	1.9649	0.08199	0.033^*^
CKT vs. CKS	2.1274	0.08817	0.025^*^

CKT: crop rotation-tolerance, CKS: crop rotation-sensitive, 7T: 7year-continuous cropping-tolerant, 7S: 7year-continuous cropping-sensitive, 36T: 36year-continuous cropping-tolerant, 36S: 36year-continuous cropping-sensitive.

* *p*<0.05;

** *p*<0.01;

*** *p*<0.001.

### Specific Microbiomes of Two Soybean Genotypes in Different Cropping Systems

**Figure 3:**
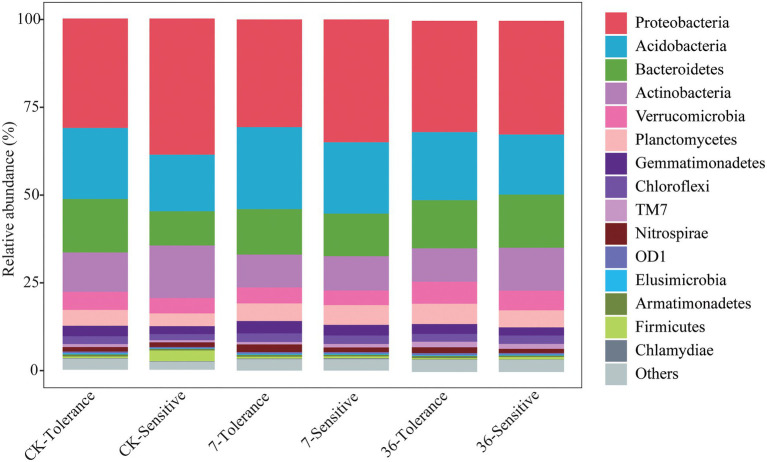
Relative abundance of the phylum of different treatments.

**Figure 4:**
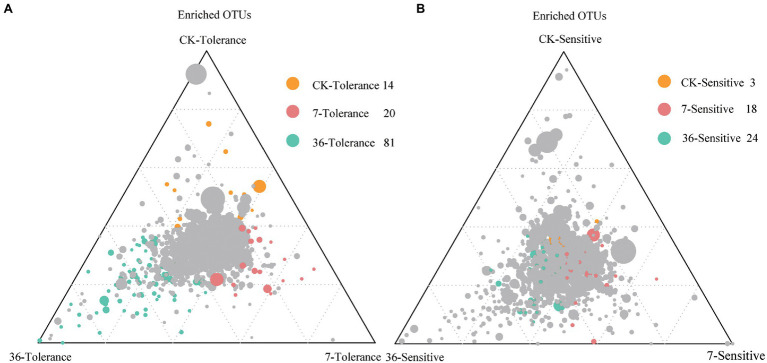
Ternary plots depicting compartments showing the distributions of community differentiation among different cropping systems of tolerant **(A)** and sensitive **(B)** genotypes. The position of each circle depends on its contribution to the total relative abundance. Colored circles represent OTUs that enriched in different cropping systems, which the green for the rotation cropping system; red and green for 7- and 36-year continuous cropping systems, respectively. The gray circles represent the OTUs that are not significantly enriched within a given range.

Proteobacteria, Bacteroidetes, Firmicutes, Acidobacteria, Actinobacteria, and Verrucomicrobia were the most abundant bacterial phyla living in the rhizosphere across all treatments, accounting for 80.57–82.98% of the whole community (Figure 3; Supplementary Table S1). Analysis of the phylum abundance with two-way ANOVA (Supplementary Table S1) showed that 10, 7, and 10 phyla were significantly (*p*<0.05) affected by cropping systems. In addition, they were significantly (*p*<0.05) affected by cropping systems, soybean genotypes, and their interaction (Supplementary Table S1). Moreover, the response to continues cropping between the two genotypes was different. For instance, the relative abundances of Acidobacteria were significantly increased in the 7-year continuous cropping field but decreased relative abundances of both soybean genotypes in the 36-year continuous cropping field, and with a higher relative abundance for the sensitive genotypes than the tolerant genotypes. The relative abundances of Proteobacteria significantly decreased and then increased in these cropping systems for the tolerant genotypes, while it continuously decreased for the sensitive genotypes. At the genus level, 16, 20, and 22 genera were significantly (*p*<0.05) affected by cropping systems, soybean genotypes, and their interaction, respectively (Table 3). Among them, some genera in different genotypes respond differently to continuous cropping. For example, *Pseudomonas*, which belong to Proteobacteria, in tolerant genotype rhizosphere increased and then decreased with continuous cropping time, while an opposite trend was observed in the sensitive genotypes. However, there are also some genera that respond to continuous cropping in a consistent trend between the two genotypes. For instance, *Streptomyces* and *Bacillus*, which belong to Actinobacteria and Firmicutes, respectively, decreased in the 7-year continuous cropping field but increased in the 36-year continuous cropping field, with higher relative abundances in the sensitive genotypes. A linear model analysis was used to identify bacterial OTUs significantly enriched in tolerant and sensitive genotypes of rhizosphere soil in the crop rotation system, 7-year continuous cropping system and 36-year continuous cropping system. For the tolerant genotypes, OTUs belonging to *Chitinophaga*, *Nitrospira*, and *Saprospirales* dominated in the rhizospheres of the crop rotation system, 7-year continuous cropping system and 36-year continuous cropping system, respectively (Figure 4A). For the sensitive genotypes, OTUs belonging to *Phyllobacteriaceae*, *Comamonadaceae*, and *Cytophagaceae* dominated in the rhizospheres of the crop rotation system, 7-year continuous cropping system and 36-year continuous cropping system, respectively (Figure 4B). More detailed information is available in Supplemental files (Supplementary Table S2).

**Table 3:** Cropping systems, soybean genotypes, and their interactive effects on the relative abundance of bacteria at genus level.

		Read numbers in each genus						ANOVA (*p*-values)			
Phylum	Genus	CKT	CKS	7T	7S	36T	36S	Year	Genotype	Year*Genotype	LSD
Bacteroidetes	Chryseobacterium	3.29±7.93	0.03±0.03	0.02±0.01	0.03±0.04	0.81±2.05	0.02±0.02	0.231	0.097	0.118	2.727
Proteobacteria	Acinetobacter	3.78±5.14	3.87±6.67	2.24±6.4	6.45±11.2	3.97±5.4	2.13±5.11	0.811	0.619	0.699	5.692
Proteobacteria	Pseudomonas	0.8±0.55	7.08±6.62	1±0.5	0.8±0.43	0.44±0.41	1.5±1.64	**0.003** ^**^	**0.004** ^**^	**<0.001** ^***^	2.291
Actinobacteria	Arthrobacter	2.45±0.9	2.23±1.71	1.67±0.9	2.32±0.84	0.71±0.39	2.61±2.93	0.345	**0.04** ^*^	**0.034** ^*^	1.245
Actinobacteria	Renibacterium	2.71±1.16	5.38±5.23	2.59±1.37	2.7±1.14	1.93±1.83	3.48±2.77	0.148	**0.028** ^*^	**0.043** ^*^	2.179
Proteobacteria	Agrobacterium	1.13±0.37	2.44±1.54	0.87±0.3	1.15±0.33	1.22±0.98	1.82±1.34	**0.035** ^*^	**0.003** ^**^	**0.001** ^**^	0.778
Actinobacteria	Aeromicrobium	0.33±0.13	0.26±0.18	0.22±0.06	0.28±0.15	0.78±1.03	0.66±0.35	**<0.001** ^***^	0.753	**0.012** ^**^	0.3746
Firmicutes	Bacillus	0.27±0.15	2.63±2.58	0.18±0.07	0.31±0.32	0.18±0.12	0.33±0.38	**0.001** ^***^	**0.006** ^**^	**<0.001** ^***^	0.876
Actinobacteria	Streptomyces	0.35±0.18	2.15±2.7	0.14±0.06	0.14±0.04	0.34±0.19	0.4±0.2	**0.006** ^**^	**0.039** ^*^	**<0.001** ^***^	0.905
Proteobacteria	Thermomonas	1.05±0.23	0.99±0.59	1.27±0.23	1.07±0.34	0.75±0.16	1.1±0.41	0.071	0.733	**0.026** ^*^	0.2884
Proteobacteria	Sphingobium	0.04±0.02	0.03±0.03	0.02±0.01	0.01±0.01	0.06±0.05	0.53±1.1	0.074	0.174	**0.042** ^*^	0.3666
Bacteroidetes	Flavisolibacter	1.14±0.41	0.85±0.41	1.52±0.86	1.03±0.17	1.31±0.32	1.2±0.36	0.102	**0.011** ^*^	**0.022** ^*^	0.3866
Verrucomicrobia	Opitutus	0.59±0.28	0.44±0.1	0.53±0.22	0.49±0.16	0.8±0.49	0.67±0.41	**0.017** ^*^	0.161	0.062	0.2521
Proteobacteria	Bradyrhizobium	0.47±0.24	0.68±0.25	0.31±0.17	0.62±0.51	0.49±0.2	0.4±0.26	0.302	**0.045** ^*^	**0.034** ^*^	0.2393
Verrucomicrobia	DA101	0.31±0.21	0.45±0.36	0.28±0.19	0.13±0.05	0.51±0.26	0.17±0.09	**0.045** ^*^	**0.05** ^*^	**<0.001** ^***^	0.18
Nitrospirae	Nitrospira	0.57±0.36	0.75±0.28	1.23±0.68	0.64±0.34	0.96±0.22	0.62±0.36	0.112	**0.017**	**<0.001** ^***^	0.328
Bacteroidetes	Adhaeribacter	0.65±0.29	0.42±0.3	0.76±0.48	0.79±0.32	0.62±0.33	0.7±0.28	0.057	0.633	0.121	0.2773
Proteobacteria	Azohydromonas	0.53±0.17	0.57±0.23	0.71±0.25	0.71±0.18	0.69±0.22	0.67±0.41	0.064	0.98	0.351	0.2074
Proteobacteria	Polaromonas	0.58±0.37	1.28±0.75	0.38±0.18	0.54±0.29	0.44±0.18	0.57±0.34	**<0.001** ^***^	**0.004** ^**^	**<0.001** ^***^	0.3264
Actinobacteria	Rubrobacter	0.69±0.26	0.58±0.17	0.73±0.22	0.64±0.23	0.57±0.18	0.4±0.17	**0.005** ^**^	**0.018** ^*^	**0.004** ^**^	0.1701
Proteobacteria	Steroidobacter	0.57±0.17	0.52±0.12	0.55±0.07	0.62±0.18	0.7±0.15	0.63±0.26	0.065	0.664	0.169	0.1378
Bacteroidetes	Flavobacterium	0.29±0.2	0.44±0.6	0.37±0.17	0.56±0.49	0.83±0.89	0.74±0.32	**0.014** ^*^	0.518	0.078	0.4155
Proteobacteria	Balneimonas	0.4±0.1	0.46±0.12	0.45±0.2	0.45±0.08	0.38±0.17	0.34±0.22	0.129	0.834	0.391	0.129
Verrucomicrobia	Luteolibacter	0.26±0.12	0.47±0.18	0.26±0.17	0.3±0.09	0.29±0.3	0.47±0.47	0.361	**0.025** ^*^	0.128	0.2088
Planctomycetes	Planctomyces	0.34±0.09	0.22±0.1	0.31±0.06	0.36±0.1	0.36±0.2	0.34±0.15	0.175	0.309	0.071	0.1025
Proteobacteria	Pseudoxanthomonas	0.13±0.07	0.09±0.15	0.12±0.09	0.5±0.44	0.64±0.35	0.44±0.3	**<0.001** ^***^	0.57	**<0.001** ^***^	0.2208
Bacteroidetes	Niastella	0.26±0.12	0.37±0.14	0.26±0.11	0.24±0.06	0.25±0.11	0.37±0.14	0.137	**0.013** ^*^	**0.009** ^*^	0.0944
Bacteroidetes	Dyadobacter	0.2±0.08	0.32±0.19	0.24±0.23	0.3±0.16	0.2±0.08	0.42±0.31	0.691	**0.005** ^*^	0.051	0.1583
Bacteroidetes	Pontibacter	0.26±0.11	0.2±0.16	0.26±0.1	0.21±0.07	0.16±0.04	0.31±0.5	0.992	0.839	0.672	0.1837
Proteobacteria	Variovorax	0.23±0.11	0.3±0.16	0.16±0.06	0.19±0.07	0.15±0.05	0.35±0.49	0.364	0.062	0.186	0.1788
Verrucomicrobia	Chthoniobacter	0.2±0.11	0.12±0.06	0.19±0.1	0.23±0.1	0.28±0.16	0.28±0.21	**0.009** ^**^	0.685	**0.031** ^*^	0.1072
Armatimonadetes	Fimbriimonas	0.21±0.11	0.15±0.08	0.18±0.05	0.17±0.05	0.22±0.1	0.15±0.09	0.957	**0.025** ^*^	0.281	0.06785
Bacteroidetes	Pedobacter	0.14±0.15	0.13±0.09	0.15±0.13	0.16±0.11	0.18±0.14	0.27±0.18	0.063	0.357	0.156	0.1117
Bacteroidetes	Chitinophaga	0.16±0.16	0.41±0.41	0.05±0.03	0.05±0.02	0.08±0.04	0.25±0.16	**<0.001** ^***^	**0.008** ^*^	**<0.001** ^***^	0.157
Proteobacteria	Nitrosovibrio	0.13±0.11	0.15±0.16	0.19±0.1	0.3±0.38	0.08±0.13	0.12±0.11	**0.028** ^*^	0.246	0.104	0.1569
Bacteroidetes	Segetibacter	0.14±0.06	0.11±0.12	0.17±0.16	0.14±0.06	0.23±0.09	0.17±0.09	0.041	0.116	0.095	0.0853
Proteobacteria	Afifella	0.14±0.04	0.12±0.06	0.14±0.03	0.12±0.04	0.15±0.06	0.27±0.51	0.313	0.593	0.536	0.1733
Proteobacteria	Aquicella	0.16±0.07	0.11±0.04	0.17±0.07	0.12±0.04	0.14±0.06	0.17±0.1	0.698	0.116	0.113	0.05423
Proteobacteria	Pedomicrobium	0.15±0.04	0.13±0.05	0.13±0.04	0.12±0.04	0.16±0.04	0.13±0.05	0.271	**0.038** ^*^	0.212	0.03692
Actinobacteria	Phycicoccus	0.15±0.05	0.28±0.18	0.08±0.02	0.08±0.03	0.09±0.03	0.11±0.06	**<0.001** ^***^	**0.046** ^*^	**<0.001** ^***^	0.06679
Planctomycetes	Gemmata	0.15±0.07	0.12±0.11	0.1±0.05	0.15±0.07	0.17±0.15	0.11±0.07	0.79	0.659	0.368	0.0759
Firmicutes	Paenibacillus	0.06±0.03	0.27±0.18	0.06±0.03	0.12±0.11	0.04±0.01	0.14±0.25	0.15	**<0.001** ^***^	**0.001**	0.1105
Actinobacteria	Kribbella	0.1±0.05	0.22±0.17	0.04±0.02	0.05±0.03	0.12±0.08	0.12±0.05	**<0.001** ^***^	0.082	**<0.001** ^***^	0.0692
Bacteroidetes	Lacibacter	0.1±0.03	0.1±0.08	0.1±0.08	0.1±0.05	0.1±0.05	0.11±0.06	0.959	0.693	0.995	0.0503

CKT: crop rotation-tolerance, CKS: crop rotation-sensitive, 7T: 7year-continuous cropping-tolerant, 7S: 7year-continuous cropping-sensitive, 36T: 36year-continuous cropping-tolerant, 36S: 36year-continuous cropping-sensitive. P-values less than 0.05 were indicated in bold letters.

* *p*<0.05;

** *p*<0.01;

*** *p*<0.001.

Correlations between rhizosphere soil microbiota with higher relative abundance and pH, soybean cyst nematode, and yield were obtained *via* Pearson’s correlation analysis (Supplementary Figure S4). Soil pH was positively correlated with *Acinetobacter* and *Bradyrhizobium*, while it was negatively correlated with other genera. Cyst nematode was negatively correlated with *Thermomonas*, *Flavisolibacter*, and *Opitutus*, while it was positively correlated with Pseudomonas, *Renibacterium*, *Agrobacterium*, *Bacillus*, and Streptomyces. Moreover, the yield was positively correlated with *Sphingobium*, *Flavisolibacter*, and *Opitutus*.

### Core Microbiome and Co-occurrence Network

**Figure 5:**
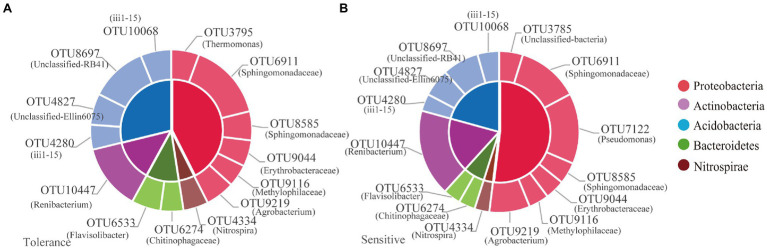
The relative abundance of the core microbiome for tolerant **(A)** and sensitive **(B)** genotypes.

Among the 12,436OTUs, we found that tolerant and sensitive genotypes shared almost the same core microorganisms, such as OTU3795 (*Thermomonas*), OTU6911 (*norank_Sphingomonadaceae*), OTU8585 (*norank*_*Sphingomonadaceae*), OTU9044 (norank_*Erythrobacteraceae*), OTU9116 (*norank_Methylophilaceae*), OTU9219 (*Agrobacterium*), OTU4334 (*Nitrospira*), OTU6274 (*norank_Chitinophagaceae*), OTU6533 (*Flavisolibacter*), OTU10447 (*Renibacterium*), OTU4280 (*norank_ii1-15*), OTU4827 (*norank_Ellin6075*), OTU8697 (*norank_RB41*), and OTU10068 (*norank_ii1-15*; Figure 5). Moreover, OTU7122 (*Pseudomonas*) was the core species specific to the sensitive genotypes (Figure 5B). The relative abundances of the total core OTUs of tolerant and sensitive Proteobacteria were 7.77% and 11.48, respectively (Supplementary Table S3).

**Figure 6:**
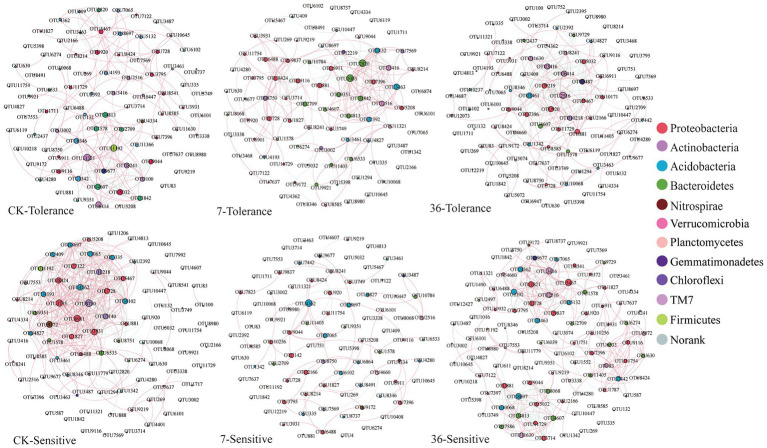
Network analysis of the rhizosphere bacterial community from different treatments. Nodes represent OTUs coded by phyla and are scaled by the number of connections (node degree). Connections are plotted at *r*>0.8 (positive correlation, red) or *r*<−0.8 (negative correlation, blue) and *p*<0.05.

**Figure 7:**
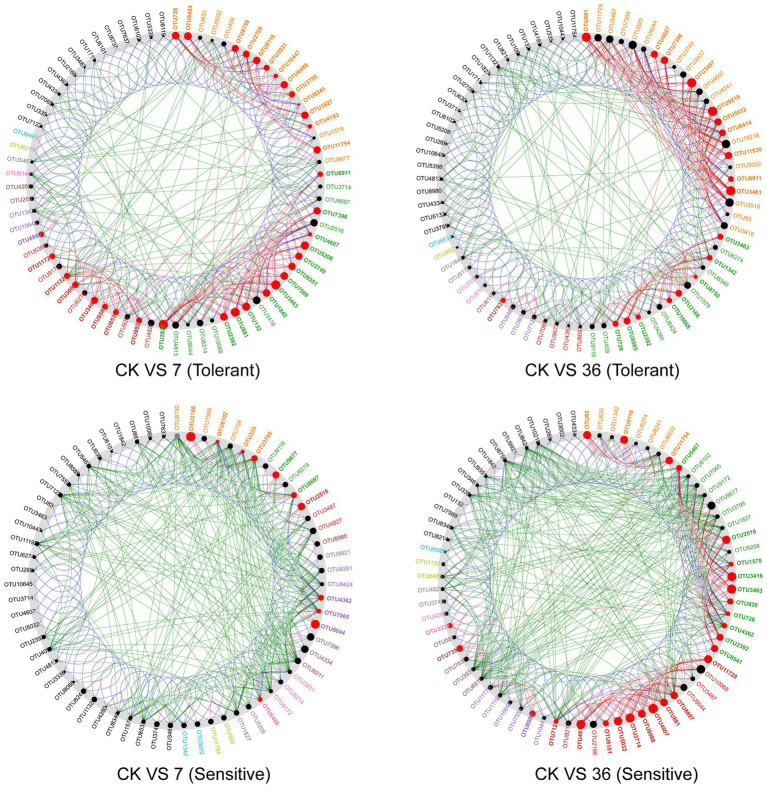
A common sub-network view with the “driver” node highlighted. All nodes belonging to the same community have been randomly assigned a similar color. Gray OTU nodes represent nodes that are present in both cases, but interact directly with the common subnetwork in either the case or control group. The size of the nodes was proportional to their NESH scores, and a node was colored red if its spacing between the control and study groups increased. Large and red nodes are particularly important “drivers.” CK VS 7 (36; tolerant or sensitive), network comparison between soybean-corn rotations and 7 (36) years continuous cropping for tolerant or sensitive genotype.

Using the combined 16S rRNA data of the two soybean genotypes grown in different cropping systems, the co-occurrence network in the rhizosphere showed marked differences between the treatments (Figure 6; Supplementary Table S4). For the tolerant genotype, the number of negative correlations and modularity (M) increased with continuous cropping years, suggesting that there were more coupling, cooperation, and exchange events among the dominant bacterial genera. Moreover, there was no significant change in the average clustering coefficient (avgCC) or average degree (avgK). For the sensitive genotype, the modularity (M) increased with continuous cropping years. However, the number of negative correlations, average clustering coefficient (avgCC), and average degree (avgK) increased in the 7-year continuous cropping systems field but decreased in the 36-year continuous cropping systems field. The keystone species of the bacterial network in the rhizosphere were identified by calculating node degree, closeness centrality, and betweenness centrality for all nodes in the network (Supplementary Table S5). In general, OTU11321 (*Luteolibacter*), OTU6533 (*Flavisolibacter*), OTU1003 (*Chitinophagaceae*), OTU4362 (*norank_RB41*), OTU881 (*Balneimonas*), and OTU3461 (*norank_Ellin6075*) were identified as keystone species for the two genotypes grown in the different cropping systems. Using NetShift analysis, the common sub-network plot showed the “driver microbes” were OTU2820 (*Unclassified Chitinophagaceae*), OTU881 (*Unclassified oc28*), and OTU3463 (*Unclassified Acidobacteria-6*) for the network between rotations and 7years continuous cropping system, and OTU881 (*Unclassified oc28*), OTU3487 (*Unclassified Gemm-1*), OTU9219 (*Agrobacterium*), and OTU3461 (*Unclassified Ellin6075*) for the network between rotations and 7years continuous cropping system. For the sensitive soybean genotype, the “drivers microbes” were OTU2166 (*Unclassified Piscirickettsiaceae*) and OTU9044 (*Unclassified Erythrobacteraceae*) for the network between rotations and 7years continuous cropping system, and OTU4813 (*Unclassified Saprospiraceae*), OTU3714 (*Unclassified Oxalobacteraceae*), OTU4607 (*Unclassified Cytophagaceae*), OTU3463 (*Unclassified Acidobacteria-6*), and OTU3416 (*Unclassified OPB35*) for the network between rotations and 7years continuous cropping system (Figure 7).

## Discussion

Our initial hypotheses were that soybean with a tolerant genotype would have higher rhizosphere bacterial diversity than soybean with a sensitive genotype, and tolerant soybean would recruit microorganisms that benefited continuous cropping tolerance. Our results showed that there was no significant difference in microbial diversity between the two genotypes soybean in all cropping systems. In addition, our second hypothesis was tested: tolerant genotypes recruited specific microorganisms that may help soybean mitigate soil-borne diseases. Short period of continuous cropping is unfavorable for soybean, but long periods of continuous cropping will mitigate this unfavorability. Moreover, different genotypes of soybean respond differently to continuous cropping systems, and microorganisms recruited by tolerant soybeans might play an important role in mitigating the adverse effects of continuous cropping.

In the present study, we found that there was no significant difference in bacterial community diversity among the treatments. Previous studies have shown that an increase in crop type could lead to an increase in soil microbial diversity. Liu et al. (2020) found that rhizosphere bacterial diversity was reduced with continuous cropping of soybean compared to soybean-corn rotation cropping systems. In addition, Zhu et al. (2017) and Liu et al. (2020) found that soil bacterial diversity increased with increasing years of continuous cropping. However, Li et al. (2010) found no significant difference in soil microbial diversity between the soil of continuous soybean and soybean-corn rotations. This might be attributed to the different soil pH in these studies, as Liu et al. conducted their research on acidic soil, while our study was conducted on neutral soil. Changes in soil pH and other physical and chemical properties were significantly associated with microbial diversity, and this variation was related to changes in root secretions, such as organic acids, phenols, and flavonoids, which were significantly affected by the cropping systems (Venter et al., 2016; Liu et al., 2020). For beta diversity, we found that cropping systems and soybean genotypes were the main factors that changed the bacterial communities (Adonis tests, *p*<0.05). This result is consistent with the results reported by Zhu et al. (2017) and Lian et al. (2019), who indicated that there were significant changes in soil bacterial communities in long-term and short-term continuous soybean cropping systems and in different soybean genotypes.

The relative abundance of Proteobacteria was significantly decreased in the rhizospheric soils of the continuous cropping system field compared to those of the rotation system, indicating that those bacteria were decreased with low available nutrients (Fierer et al., 2007; Li et al., 2014). The relative abundances of *Pseudomonas*, *Streptomyces*, and *Bacillus* decreased in the 7-year continuous cropping field but increased in the 36-year continuous cropping field, with a higher relative abundance in the sensitive genotypes. *Pseudomonas* can improve the solubilization of fixed soil phosphorus and applied phosphates, resulting in higher crop yields (Nautiyal, 1999). Moreover, the metabolites of *Pseudomonas* could increase amino acid secretion in alfalfa, maize, and wheat roots (Phillips et al., 2004). *Streptomyces* can help plants resist diseases such as wilt and root rot (Lian et al., 2019; Shi et al., 2020). In addition, some bacteria, such as *Thermomonas*, *Flavisolibacter*, and *Opitutus* were negatively associated with nematodes, which suggested that these bacteria might inhibit the soybean cyst nematode. Thus, based on the functions of the species mentioned above, we speculated that the changed relative abundances of these bacteria might be associated with the antagonistic activity of these taxa against plant pathogens and the improvement of soil nutrients.

We used differential OTU abundance analysis in the treatments and observed a high relative abundance of the OTUs enriched in continuous cropping systems of the tolerant genotypes that belonged to *Chitinophagaceae*, *Pseudoxanthomonas*, *Nitrospira*, and Streptomyces (Figure 4; Table 3). This finding is consistent with our hypothesis that tolerant genotypes recruited some microorganisms that play an important role in the resistance of soybeans to long-term continuous cropping. OTU4434 (*Nitrospira*), OTU751 (*Pseudoxanthomonas*), and OTU10218 (*Streptomyces*) showed higher relative abundances in the continuous cropping system than in the rotation cropping system in the present study (Figure 4; Table 3). Many studies have reported that *Nitrospira* can promote the nitrogen cycle and increase the absorption and utilization of nitrogen by plants (Zeng et al., 2014). Therefore, the higher abundances of these bacteria may improve soybean tolerance in the rhizosphere. However, the response of these genera to long-term continuous cropping warrants further investigation of their functional significance in response to different long-term continuous cropping systems.

An association network analysis was performed to gain a more integrated understanding of the bacterial community composition and compare the complexities of the networks operating in the rhizosphere soils of continuous cropping-tolerant and continuous cropping-sensitive soybean genotypes (Jiang et al., 2017). The networks of the two genotypes responded differently to continuous cropping. The modularity and negative correlations in the network of the tolerant genotype responded positively to continuous cropping, suggesting that there were more coupling, cooperation, and exchange events among the dominant bacterial genera (Coyte et al., 2015). For the sensitive genotype, the negative correlations and average degree (avgK) increased in the 7-year continuous cropping system field but decreased in the 36-year continuous cropping system field, suggesting that the instability of the sensitive genotype and short periods of continuous planting can have a detrimental effect on microbial community stability, but this effect could be alleviated with increasing periods of continuous planting. This result was consistent with a previous study, which found that a long-term continuous cropping system has similar microbial interactions to those of a healthy crop rotation system (Liu et al., 2020). Additionally, the important microbial taxa in the networks which serve as “drivers” from the rotations to short or long continue cropping were different between the two soybean genotypes, which indicated that the response of rhizosphere microbial community under different soybean genotypes to short- and long-term continuous cropping was different.

In summary, short- or long-term continuous cropping had no significant effect on the rhizosphere soil bacterial alpha diversity. Short-term continuous planting increased the number of soybean cyst nematodes, while long-term continuous planting reduced these numbers, and tolerant genotypes had lower soybean cyst nematodes than sensitive genotypes. In addition, continuous cropping significantly increased potentially beneficial bacterial populations compared to rotation and short-term continuous cropping, suggesting that long-term continuous cropping of soybean shifts the microbial community into that of a healthy crop rotation system. Soybean genotypes that are tolerant might recruit some microorganisms that play an important role in the resistance of soybeans to long-term continuous cropping. However, further experiment with species isolation and verification is needed to confirm these findings. Moreover, the networks of the two genotypes responded differently to continuous cropping. The tolerant genotype responded positively to continuous cropping, while for the sensitive genotype, topology suggested that the instability of the sensitive genotype and short periods of continuous planting can have a detrimental effect, but this effect could be alleviated with increasing periods of continuous planting.

## Data Availability Statement

The datasets presented in this study can be found in online repositories. The names of the repository/repositories and accession number(s) can be found in the article/Supplementary Material.

## Author Contributions

TL, HN, and MY designed the research. MY, KY, LW, DH, and SW performed the research. TY, QS, HX, and RW analyzed the data. MY and TL analyzed the data and wrote the manuscript. All authors contributed to the article and approved the submitted version.

## Funding

This work was supported by China Agriculture Research System of MOF and MARA (CARS-04), and the Laboratory independent research projects (SKLCUSA-a201913).

## Conflict of Interest

The authors declare that the research was conducted in the absence of any commercial or financial relationships that could be construed as a potential conflict of interest.

## Publisher’s Note

All claims expressed in this article are solely those of the authors and do not necessarily represent those of their affiliated organizations, or those of the publisher, the editors and the reviewers. Any product that may be evaluated in this article, or claim that may be made by its manufacturer, is not guaranteed or endorsed by the publisher.

## Footnotes

^1^ https://web.rniapps.net/netshift/
