# Supplementary material for: Rhizosphere Soil Bacterial Communities of Continuous Cropping-Tolerant and Sensitive Soybean Genotypes Respond Differently to Long-Term Continuous Cropping in Mollisols
Source: Front Microbiol. 2021 Sep 13;12:729047. doi: 10.3389/fmicb.2021.729047 (PMC8473881; doi:10.3389/fmicb.2021.729047)
Supplement: Supplementary file 1 [file Data_Sheet_1.docx]

**Rhizosphere soil bacterial communities of continuous cropping-tolerant and sensitive soybean genotypes respond differently to long-term continuous cropping in Mollisols**

Yuan Ming^1^, Yu Taobing^2,3^, Shi Qihan^2,3^, Han Dongwei^1^, Yu Kanchao^1^, Wang Lianxia^1^, Wang Shurong^1^, Xiang Hao^4^, Wen Ronghui^5^, Nian Hai^2,3^*, Lian Tengxiang^2,3^*

^1^Qiqihar Branch of Heilongjiang Academy of Agricultural Sciences, Qiqihar, Heilongjiang, China

^2^The State Key Laboratory for Conservation and Utilization of Subtropical Agro-bioresources, South China Agricultural University, Guangzhou, Guangdong, China

^3^The Key Laboratory of Plant Molecular Breeding of Guangdong Province, College of Agriculture, South China Agricultural University, Guangzhou, Guangdong, China

^4^ Institute of Hydrobiology, Chinese Academy of Sciences, Wuhan, China

^5^ The State Key Laboratory for Conservation and Utilization of Subtropical Agro-bioresources, College of Life Science and Technology, Guangxi University, Nanning, China

***Corresponding author1:** Tengxiang Lian

**Corresponding address: No.**483 Wushan Road, Guangzhou, Guangdong, 510642, China.

**Tel:** +86 02085288024

Fax: +86 02085288024

E-mail address: liantx@scau.edu.cn

***Corresponding author2:** Hai Nian

**Corresponding address: No.**483 Wushan Road, Guangzhou, Guangdong, 510642, China.

**Tel:** +86 02085288024

Fax: +86 02085288024

**E-**mail address: [hnian@scau.edu.cn](mailto:hnian@scau.edu.cn)


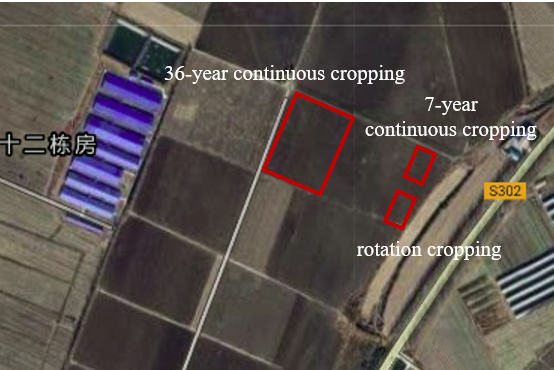


Figure S1. Distribution map of the experimental design.


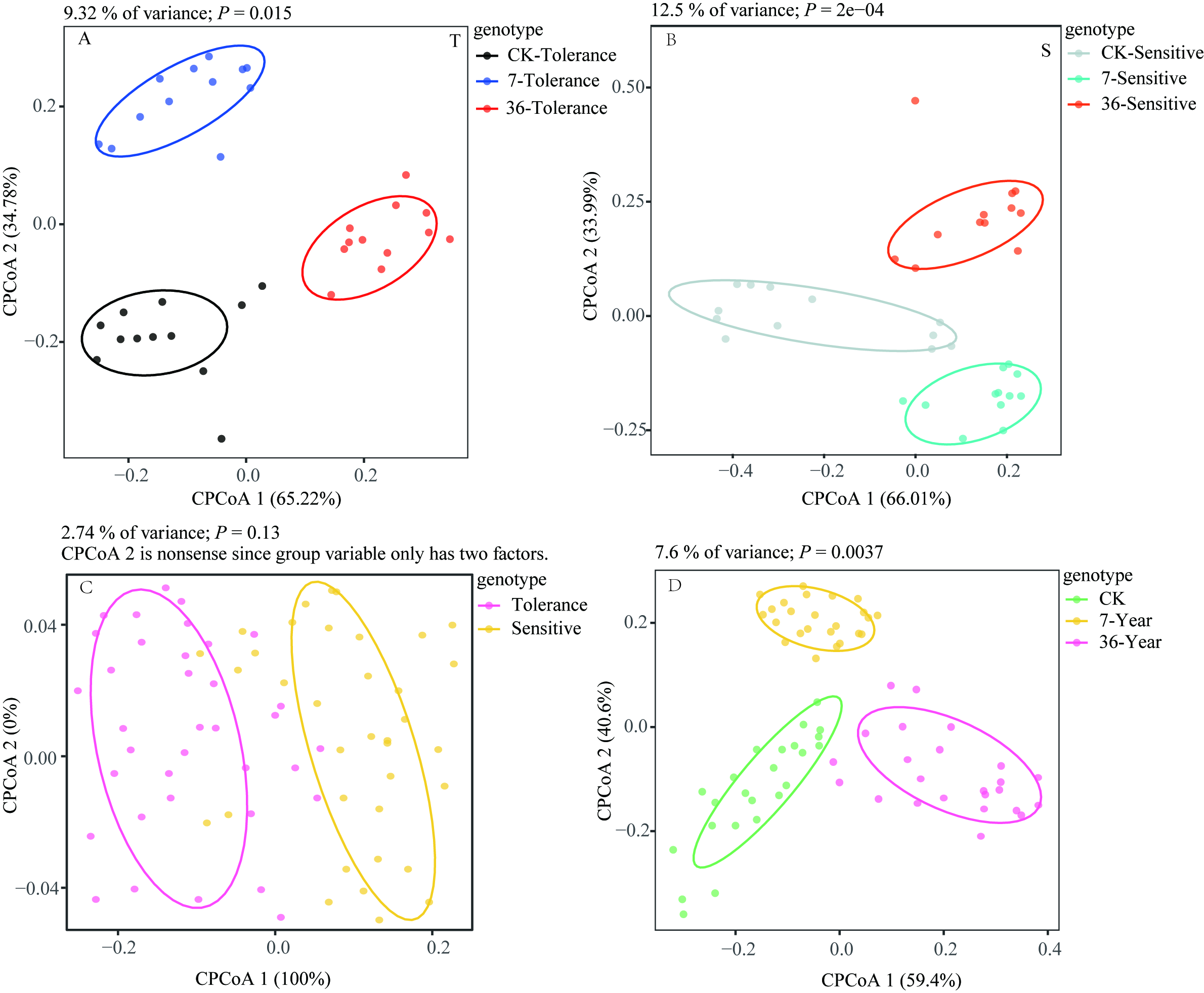


Figure S2. Constrained Principal coordinate analysis (CPCoA) based on Bray-Curtis dissimilarities of 16S rRNA diversity in the rhizosphere of the tolerant (A) (PERMANOVA, *P* = 0.015) and sensitive (B) (PERMANOVA, *P* = 0.0.0002) genotype in different cropping systems. CPCoA of the two genotypes (C) (PERMANOVA, *P* = 0.013), and three cropping systems (D) (PERMANOVA, *P* = 0.0037). CKT: crop rotation-tolerance, CKS: crop rotation-sensitive, 7T: 7 year-continuous cropping-tolerant, 7S: 7 year-continuous cropping-sensitive, 36T: 36 year-continuous cropping-tolerant, 36S: 36 year-continuous cropping-sensitive.


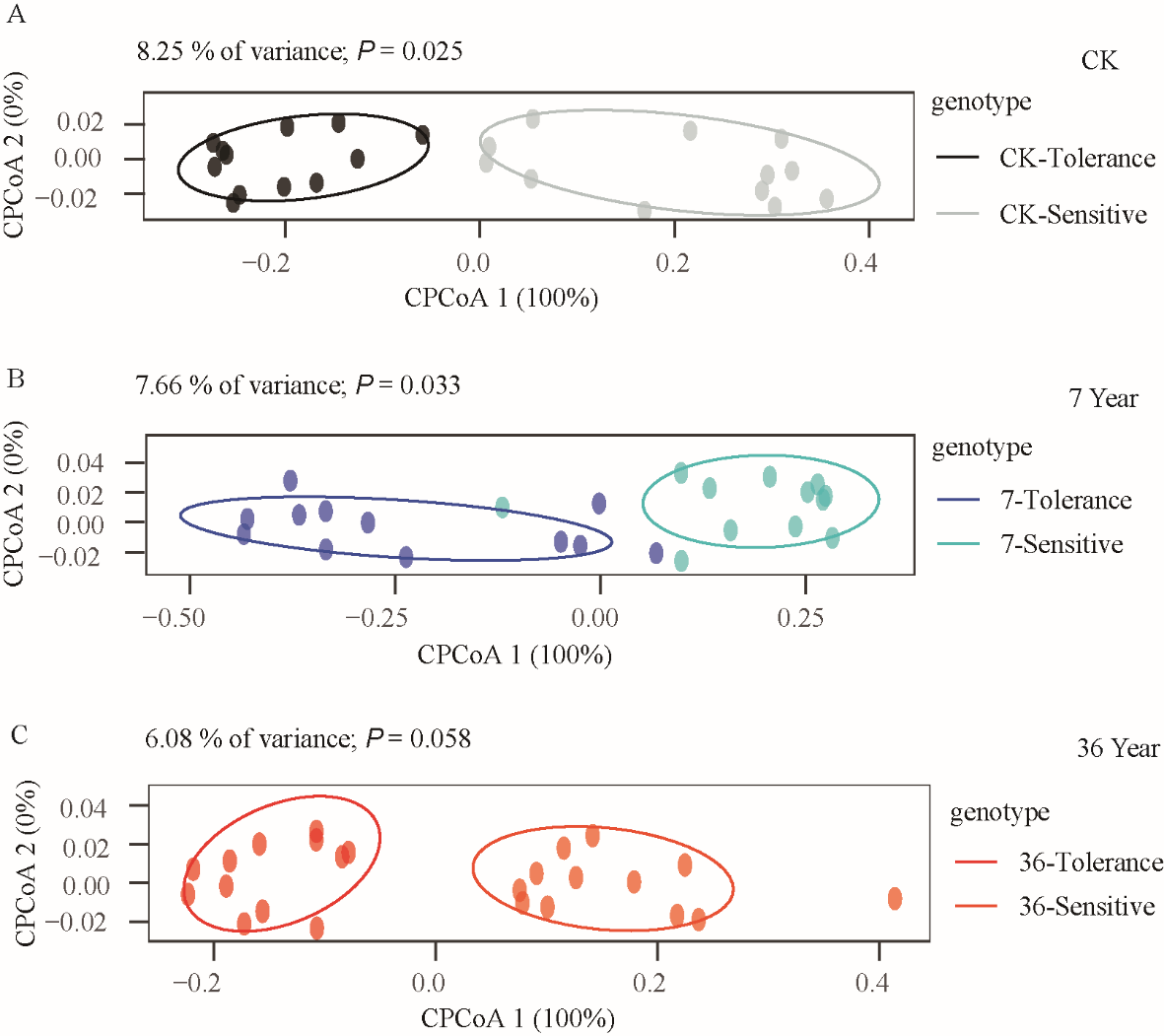


Figure S3. Constrained Principal coordinate analysis (PCoA) based on Bray-Curtis dissimilarities of 16S rRNA diversity in the rhizosphere of the two soybean genotypes (PERMANOVA, *P* = 0.001)


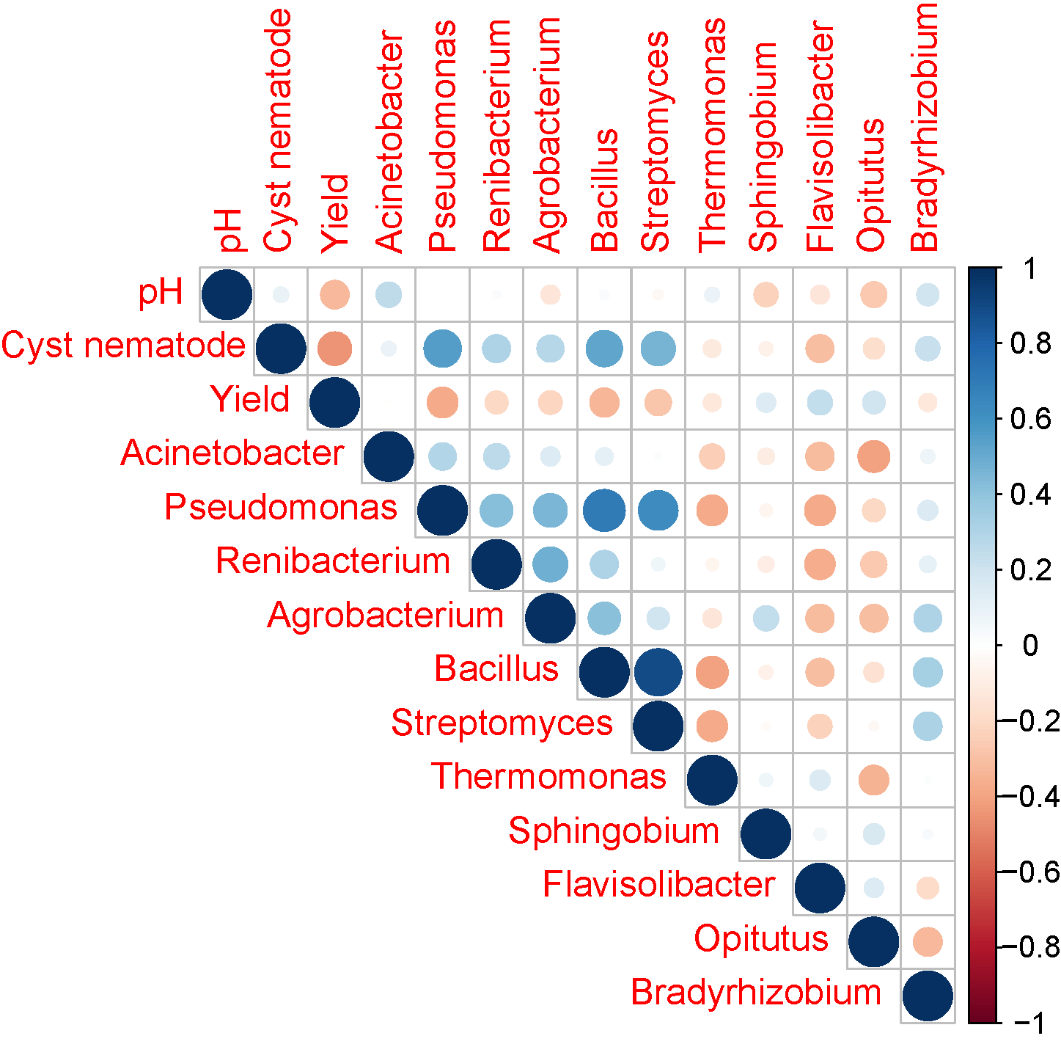


Fig S4 Correlation analysis between pH, cyst nematode, yield and the genera with higher relative abundance.

Table S1. Cropping systems, soybean genotypes and their interactive effects on the relative abundance of bacteria at phylum level.

|  | Read numbers in each genus | | | | | | ANOVA(*P*-values) | | |  |
| --- | --- | --- | --- | --- | --- | --- | --- | --- | --- | --- |
| Phylum | CKT | CKS | 7T | 7S | 36T | 36S | Cropping systems | Genotype | Cropping systems*Genotype | LSD |
| Proteobacteria | 31.17±5.51 | 38.77±7.76 | 30.66±6.3 | 34.96±6.87 | 31.69±7.14 | 32.37±6.22 | 0.333 | **0.011** | **0.037** | 5.467 |
| Bacteroidetes | 15.24±6.18 | 9.75±2.4 | 12.93±3.42 | 12.12±2.3 | 13.7±2.92 | 15.13±2.52 | 0.161 | 0.083 | **0.004** | 2.964 |
| Acidobacteria | 20.22±4.26 | 16.12±5.62 | 23.37±2.35 | 20.35±3.73 | 19.39±3.28 | 17.13±3.88 | **0.004** | **0.003** | **<.001** | 3.271 |
| Actinobacteria | 11.19±2.76 | 14.98±5.91 | 9.36±1.93 | 9.76±1.75 | 9.53±2.81 | 12.23±4.86 | **0.009** | **0.016** | **0.002** | 3.005 |
| Verrucomicrobia | 5.16±2.16 | 4.37±1.74 | 4.56±1.89 | 4.17±0.88 | 6.26±3.54 | 5.61±2.27 | **0.045** | 0.259 | 0.184 | 1.827 |
| Planctomycetes | 4.52±1.05 | 3.62±1.89 | 5.03±0.7 | 5.63±1.18 | 5.8±3.33 | 4.85±1.69 | **0.031** | 0.366 | 0.065 | 1.51 |
| Gemmatimonadetes | 2.98±0.48 | 2.24±0.96 | 3.51±0.48 | 3.03±0.61 | 2.8±0.41 | 2.31±0.89 | **0.001** | **0.002** | **<.001** | 0.5507 |
| Chloroflexi | 2.22±0.63 | 1.74±0.68 | 2.44±0.47 | 2.42±0.67 | 2.21±0.39 | 2.39±0.56 | **0.032** | 0.451 | **0.046** | 0.4757 |
| Firmicutes | 0.49±0.19 | 3.05±2.76 | 0.54±0.24 | 0.56±0.48 | 0.38±0.32 | 0.71±0.73 | **0.003** | **0.005** | **<.001** | 0.974 |
| TM7 | 0.86±0.45 | 0.63±0.24 | 0.7±0.26 | 0.99±0.35 | 1.62±0.69 | 1.4±0.39 | **<.001** | 0.687 | **<.001** | 0.347 |
| Nitrospirae | 1.26±0.36 | 1.32±0.48 | 2.17±0.67 | 1.33±0.4 | 1.64±0.28 | 1.19±0.44 | **0.01** | **0.001** | **<.001** | 0.3723 |
| Armatimonadetes | 0.59±0.22 | 0.47±0.18 | 0.56±0.16 | 0.5±0.1 | 0.64±0.22 | 0.43±0.23 | 0.997 | **0.004** | 0.084 | 0.1559 |
| OD1 | 0.52±0.22 | 0.37±0.14 | 0.49±0.18 | 0.48±0.1 | 0.57±0.29 | 0.47±0.28 | 0.527 | 0.102 | 0.373 | 0.1766 |
| Chlamydiae | 0.11±0.06 | 0.1±0.06 | 0.15±0.15 | 0.23±0.24 | 0.07±0.04 | 0.07±0.05 | **0.004** | 0.562 | **0.02** | 0.102 |
| Elusimicrobia | 0.3±0.14 | 0.17±0.05 | 0.27±0.13 | 0.24±0.07 | 0.24±0.13 | 0.25±0.19 | 0.879 | 0.093 | 0.232 | 0.1035 |
| Others | 3.15±0.63 | 2.3±0.69 | 3.25±0.52 | 3.23±0.55 | 3.46±0.74 | 3.47±1.2 | **0.006** | 0.147 | **0.004** | 0.6189 |

Table S2 The sequence number of bacterial OTUs in the ternary plots depicting compartment.

| OTU ID | 36 year | 7 year | CK | Aver | group | rich | Phylum | genus |
| --- | --- | --- | --- | --- | --- | --- | --- | --- |
| OTU1717 | 6.42 | 6.92 | 39.83 | 17.72 | CKT | CKT | Bacteroidetes | Chitinophaga |
| OTU12069 | 4.00 | 7.75 | 22.25 | 11.33 | CKT | CKT | Bacteroidetes | norank |
| OTU9854 | 2.75 | 3.58 | 5.75 | 4.03 | CKT | CKT | Bacteroidetes | norank |
| OTU6121 | 12.17 | 25.08 | 28.08 | 21.78 | CKT | CKT | Bacteroidetes | norank |
| OTU7765 | 2.67 | 7.00 | 10.00 | 6.56 | CKT | CKT | Bacteroidetes | norank |
| OTU10645 | 59.33 | 280.92 | 389.50 | 243.25 | CKT | CKT | Actinobacteria | Arthrobacter |
| OTU3361 | 29.75 | 16.33 | 30.25 | 25.44 | CKT | CKT | Actinobacteria | norank |
| OTU9275 | 10.33 | 5.83 | 11.92 | 9.36 | CKT | CKT | Actinobacteria | norank |
| OTU9853 | 8.17 | 2.58 | 12.75 | 7.83 | CKT | CKT | Proteobacteria | norank |
| OTU9439 | 5.67 | 2.42 | 8.83 | 5.64 | CKT | CKT | Proteobacteria | norank |
| OTU7831 | 3.33 | 3.75 | 6.08 | 4.39 | CKT | CKT | Elusimicrobia | norank |
| OTU10888 | 3.25 | 6.75 | 7.58 | 5.86 | CKT | CKT | Actinobacteria | Rubrobacter |
| OTU1672 | 4.08 | 7.42 | 12.17 | 7.89 | CKT | CKT | Bacteroidetes | norank |
| OTU10706 | 1.25 | 3.67 | 4.08 | 3.00 | CKT | CKT | Proteobacteria | norank |
| OTU4334 | 271.17 | 308.75 | 158.83 | 246.25 | 7T | 7T | Nitrospirae | Nitrospira |
| OTU10784 | 41.33 | 104.92 | 33.00 | 59.75 | 7T | 7T | Bacteroidetes | norank |
| OTU6513 | 17.17 | 34.83 | 33.50 | 28.50 | 7T | 7T | Bacteroidetes | norank |
| OTU1954 | 19.08 | 34.42 | 19.17 | 24.22 | 7T | 7T | Acidobacteria | norank |
| OTU11245 | 16.92 | 37.00 | 17.58 | 23.83 | 7T | 7T | Bacteroidetes | norank |
| OTU3431 | 10.67 | 30.25 | 22.25 | 21.06 | 7T | 7T | Proteobacteria | norank |
| OTU9163 | 9.58 | 22.33 | 17.00 | 16.31 | 7T | 7T | Acidobacteria | norank |
| OTU7818 | 6.33 | 15.25 | 6.92 | 9.50 | 7T | 7T | Acidobacteria | norank |
| OTU7236 | 8.25 | 12.25 | 5.08 | 8.53 | 7T | 7T | Gemmatimonadetes | norank |
| OTU10873 | 4.17 | 9.75 | 8.58 | 7.50 | 7T | 7T | Acidobacteria | norank |
| OTU8779 | 4.92 | 7.42 | 2.42 | 4.92 | 7T | 7T | Proteobacteria | norank |
| OTU6868 | 2.17 | 6.08 | 5.33 | 4.53 | 7T | 7T | Proteobacteria | norank |
| OTU4049 | 1.25 | 7.67 | 2.50 | 3.81 | 7T | 7T | Acidobacteria | norank |
| OTU10370 | 2.83 | 5.08 | 2.83 | 3.58 | 7T | 7T | Gemmatimonadetes | norank |
| OTU9593 | 1.33 | 6.17 | 3.00 | 3.50 | 7T | 7T | Acidobacteria | norank |
| OTU10905 | 0.58 | 7.08 | 2.58 | 3.42 | 7T | 7T | norank | norank |
| OTU998 | 2.17 | 5.33 | 2.42 | 3.31 | 7T | 7T | Planctomycetes | norank |
| OTU10687 | 2.25 | 6.00 | 1.67 | 3.31 | 7T | 7T | Proteobacteria | norank |
| OTU2856 | 1.33 | 5.00 | 3.33 | 3.22 | 7T | 7T | Chlamydiae | norank |
| OTU4793 | 1.58 | 5.83 | 2.00 | 3.14 | 7T | 7T | Planctomycetes | norank |
| OTU6274 | 316.00 | 192.83 | 184.33 | 231.06 | 36T | 36T | Bacteroidetes | norank |
| OTU751 | 181.17 | 29.92 | 35.50 | 82.19 | 36T | 36T | Proteobacteria | Pseudoxanthomonas |
| OTU10218 | 109.08 | 37.50 | 74.83 | 73.81 | 36T | 36T | Actinobacteria | Streptomyces |
| OTU11405 | 75.42 | 68.67 | 37.75 | 60.61 | 36T | 36T | Bacteroidetes | norank |
| OTU9153 | 46.67 | 39.58 | 22.67 | 36.31 | 36T | 36T | Bacteroidetes | norank |
| OTU9729 | 76.75 | 14.83 | 11.58 | 34.39 | 36T | 36T | Bacteroidetes | Flavobacterium |
| OTU4851 | 51.00 | 24.08 | 26.83 | 33.97 | 36T | 36T | Proteobacteria | norank |
| OTU11350 | 51.67 | 26.42 | 22.50 | 33.53 | 36T | 36T | Bacteroidetes | Segetibacter |
| OTU4401 | 40.83 | 14.33 | 30.25 | 28.47 | 36T | 36T | Actinobacteria | Kribbella |
| OTU10166 | 34.67 | 20.17 | 20.75 | 25.19 | 36T | 36T | Chlorobi | norank |
| OTU4000 | 32.75 | 20.42 | 20.92 | 24.69 | 36T | 36T | Acidobacteria | norank |
| OTU5793 | 42.67 | 4.08 | 20.83 | 22.53 | 36T | 36T | Actinobacteria | norank |
| OTU6396 | 34.58 | 9.50 | 23.42 | 22.50 | 36T | 36T | Actinobacteria | Mycobacterium |
| OTU7081 | 25.92 | 14.58 | 11.42 | 17.31 | 36T | 36T | Bacteroidetes | norank |
| OTU11530 | 23.75 | 8.58 | 15.75 | 16.03 | 36T | 36T | Acidobacteria | norank |
| OTU1371 | 28.58 | 10.33 | 7.67 | 15.53 | 36T | 36T | Proteobacteria | Steroidobacter |
| OTU6175 | 33.08 | 5.92 | 6.00 | 15.00 | 36T | 36T | Verrucomicrobia | DA101 |
| OTU4402 | 24.50 | 11.00 | 8.92 | 14.81 | 36T | 36T | Proteobacteria | norank |
| OTU7816 | 21.33 | 11.75 | 10.67 | 14.58 | 36T | 36T | Chloroflexi | norank |
| OTU1999 | 25.50 | 8.33 | 8.75 | 14.19 | 36T | 36T | Planctomycetes | norank |
| OTU5851 | 20.17 | 10.58 | 9.42 | 13.39 | 36T | 36T | Armatimonadetes | norank |
| OTU9694 | 23.58 | 6.92 | 8.92 | 13.14 | 36T | 36T | Acidobacteria | norank |
| OTU9154 | 19.83 | 7.25 | 9.75 | 12.28 | 36T | 36T | Planctomycetes | norank |
| OTU8310 | 18.25 | 10.75 | 7.67 | 12.22 | 36T | 36T | Bacteroidetes | norank |
| OTU8418 | 17.67 | 5.58 | 13.25 | 12.17 | 36T | 36T | Bacteroidetes | norank |
| OTU2261 | 17.08 | 9.83 | 9.00 | 11.97 | 36T | 36T | Verrucomicrobia | DA101 |
| OTU103 | 23.25 | 5.75 | 4.83 | 11.28 | 36T | 36T | Verrucomicrobia | DA101 |
| OTU6785 | 32.08 | 0.00 | 0.25 | 10.78 | 36T | 36T | TM7 | norank |
| OTU2022 | 14.50 | 7.25 | 6.67 | 9.47 | 36T | 36T | TM7 | norank |
| OTU9325 | 14.92 | 6.92 | 6.50 | 9.44 | 36T | 36T | Bacteroidetes | norank |
| OTU12260 | 15.83 | 7.17 | 4.67 | 9.22 | 36T | 36T | Planctomycetes | norank |
| OTU5031 | 17.42 | 6.42 | 2.67 | 8.83 | 36T | 36T | FBP | norank |
| OTU2643 | 16.50 | 4.33 | 5.50 | 8.78 | 36T | 36T | Bacteroidetes | Chitinophaga |
| OTU4120 | 12.42 | 6.42 | 7.50 | 8.78 | 36T | 36T | Proteobacteria | norank |
| OTU7681 | 14.08 | 10.25 | 0.92 | 8.42 | 36T | 36T | Bacteroidetes | Flavobacterium |
| OTU7614 | 15.92 | 3.92 | 4.75 | 8.19 | 36T | 36T | Bacteroidetes | norank |
| OTU9771 | 14.83 | 5.50 | 3.92 | 8.08 | 36T | 36T | Verrucomicrobia | Ellin506 |
| OTU7203 | 14.25 | 3.83 | 6.00 | 8.03 | 36T | 36T | Actinobacteria | norank |
| OTU4937 | 15.67 | 3.42 | 4.42 | 7.83 | 36T | 36T | Verrucomicrobia | norank |
| OTU188 | 12.92 | 3.58 | 6.50 | 7.67 | 36T | 36T | Actinobacteria | norank |
| OTU6339 | 11.67 | 6.42 | 4.92 | 7.67 | 36T | 36T | Bacteroidetes | norank |
| OTU7849 | 11.42 | 7.17 | 3.58 | 7.39 | 36T | 36T | Proteobacteria | norank |
| OTU12230 | 13.75 | 5.75 | 2.50 | 7.33 | 36T | 36T | Bacteroidetes | norank |
| OTU10281 | 18.00 | 1.58 | 2.08 | 7.22 | 36T | 36T | Actinobacteria | norank |
| OTU11909 | 12.00 | 3.50 | 5.92 | 7.14 | 36T | 36T | Bacteroidetes | norank |
| OTU200 | 13.33 | 5.67 | 2.00 | 7.00 | 36T | 36T | Proteobacteria | norank |
| OTU10958 | 10.42 | 6.58 | 2.92 | 6.64 | 36T | 36T | Actinobacteria | norank |
| OTU8656 | 13.25 | 2.58 | 3.92 | 6.58 | 36T | 36T | Actinobacteria | norank |
| OTU12393 | 11.25 | 3.50 | 4.17 | 6.31 | 36T | 36T | Verrucomicrobia | norank |
| OTU2511 | 12.00 | 2.58 | 4.08 | 6.22 | 36T | 36T | TM7 | norank |
| OTU748 | 11.08 | 3.00 | 4.08 | 6.06 | 36T | 36T | Acidobacteria | norank |
| OTU6556 | 8.83 | 2.25 | 6.25 | 5.78 | 36T | 36T | Proteobacteria | norank |
| OTU1087 | 13.75 | 2.83 | 0.58 | 5.72 | 36T | 36T | Verrucomicrobia | norank |
| OTU9494 | 9.75 | 1.92 | 4.25 | 5.31 | 36T | 36T | Verrucomicrobia | norank |
| OTU9565 | 11.50 | 0.42 | 3.83 | 5.25 | 36T | 36T | Actinobacteria | Saccharothrix |
| OTU8549 | 9.67 | 3.08 | 2.50 | 5.08 | 36T | 36T | Bacteroidetes | norank |
| OTU12055 | 9.83 | 2.00 | 3.42 | 5.08 | 36T | 36T | Bacteroidetes | norank |
| OTU4443 | 10.25 | 1.75 | 2.58 | 4.86 | 36T | 36T | Proteobacteria | norank |
| OTU3813 | 12.83 | 1.00 | 0.00 | 4.61 | 36T | 36T | Proteobacteria | norank |
| OTU9971 | 10.08 | 1.58 | 1.33 | 4.33 | 36T | 36T | Verrucomicrobia | norank |
| OTU3635 | 6.42 | 5.08 | 1.42 | 4.31 | 36T | 36T | norank | norank |
| OTU5503 | 8.92 | 2.58 | 1.42 | 4.31 | 36T | 36T | Planctomycetes | norank |
| OTU9768 | 7.08 | 3.50 | 2.00 | 4.19 | 36T | 36T | Bacteroidetes | Fluviicola |
| OTU3483 | 11.08 | 0.50 | 0.67 | 4.08 | 36T | 36T | Actinobacteria | norank |
| OTU7852 | 6.33 | 2.33 | 3.50 | 4.06 | 36T | 36T | Planctomycetes | Pirellula |
| OTU4101 | 6.67 | 2.42 | 2.67 | 3.92 | 36T | 36T | Planctomycetes | norank |
| OTU3917 | 6.92 | 1.75 | 2.75 | 3.81 | 36T | 36T | Proteobacteria | norank |
| OTU3004 | 6.00 | 1.50 | 3.75 | 3.75 | 36T | 36T | FBP | norank |
| OTU5575 | 6.83 | 1.67 | 2.75 | 3.75 | 36T | 36T | Verrucomicrobia | Ellin506 |
| OTU2069 | 9.00 | 0.33 | 1.42 | 3.58 | 36T | 36T | TM7 | norank |
| OTU3375 | 5.75 | 1.33 | 3.58 | 3.56 | 36T | 36T | norank | norank |
| OTU9468 | 6.33 | 1.83 | 2.50 | 3.56 | 36T | 36T | Proteobacteria | norank |
| OTU10748 | 5.83 | 2.50 | 2.17 | 3.50 | 36T | 36T | Proteobacteria | norank |
| OTU9068 | 5.42 | 1.67 | 3.25 | 3.44 | 36T | 36T | Bacteroidetes | norank |
| OTU265 | 4.25 | 4.17 | 1.75 | 3.39 | 36T | 36T | Planctomycetes | norank |
| OTU4545 | 5.17 | 1.42 | 3.58 | 3.39 | 36T | 36T | Actinobacteria | norank |
| OTU11678 | 5.17 | 2.58 | 2.42 | 3.39 | 36T | 36T | Bacteroidetes | norank |
| OTU9288 | 8.50 | 0.92 | 0.58 | 3.33 | 36T | 36T | Actinobacteria | norank |
| OTU5651 | 6.58 | 1.33 | 1.92 | 3.28 | 36T | 36T | Actinobacteria | norank |
| OTU6550 | 5.17 | 1.08 | 2.92 | 3.06 | 36T | 36T | Bacteroidetes | norank |
| OTU2400 | 5.17 | 2.50 | 1.33 | 3.00 | 36T | 36T | Planctomycetes | norank |
| OTU269 | 158.25 | 130.58 | 158.92 | 149.25 | CKS | CKS | Proteobacteria | norank |
| OTU10164 | 15.67 | 15.25 | 15.92 | 15.61 | CKS | CKS | Acidobacteria | norank |
| OTU6705 | 4.08 | 8.17 | 8.83 | 7.03 | CKS | CKS | Acidobacteria | norank |
| OTU3931 | 140.83 | 242.08 | 225.42 | 202.78 | 7S | 7S | Proteobacteria | norank |
| OTU1827 | 92.50 | 98.50 | 79.50 | 90.17 | 7S | 7S | Proteobacteria | norank |
| OTU1455 | 31.42 | 45.75 | 24.08 | 33.75 | 7S | 7S | Nitrospirae | norank |
| OTU11245 | 33.00 | 34.50 | 26.00 | 31.17 | 7S | 7S | Bacteroidetes | norank |
| OTU6378 | 31.08 | 34.75 | 22.58 | 29.47 | 7S | 7S | Verrucomicrobia | norank |
| OTU9728 | 29.33 | 31.17 | 26.25 | 28.92 | 7S | 7S | Proteobacteria | norank |
| OTU3369 | 21.50 | 30.17 | 22.83 | 24.83 | 7S | 7S | Actinobacteria | Rubrobacter |
| OTU7681 | 20.25 | 28.92 | 0.33 | 16.50 | 7S | 7S | Bacteroidetes | Flavobacterium |
| OTU2483 | 7.75 | 14.92 | 5.67 | 9.44 | 7S | 7S | Gemmatimonadetes | norank |
| OTU5197 | 8.17 | 9.08 | 1.17 | 6.14 | 7S | 7S | Chloroflexi | norank |
| OTU2784 | 5.25 | 5.50 | 4.75 | 5.17 | 7S | 7S | Proteobacteria | Aquicella |
| OTU10370 | 4.33 | 5.58 | 3.83 | 4.58 | 7S | 7S | Gemmatimonadetes | norank |
| OTU5453 | 3.83 | 6.33 | 3.08 | 4.42 | 7S | 7S | Acidobacteria | norank |
| OTU3388 | 2.83 | 5.75 | 4.33 | 4.31 | 7S | 7S | Gemmatimonadetes | norank |
| OTU4072 | 4.75 | 5.58 | 1.67 | 4.00 | 7S | 7S | Gemmatimonadetes | norank |
| OTU1462 | 2.58 | 6.00 | 2.58 | 3.72 | 7S | 7S | Acidobacteria | norank |
| OTU1197 | 1.08 | 6.25 | 2.08 | 3.14 | 7S | 7S | Gemmatimonadetes | norank |
| OTU8133 | 3.50 | 3.67 | 2.08 | 3.08 | 7S | 7S | Gemmatimonadetes | norank |
| OTU9116 | 292.33 | 212.42 | 250.50 | 251.75 | 36S | 36S | Proteobacteria | norank |
| OTU4607 | 212.58 | 197.08 | 60.83 | 156.83 | 36S | 36S | Bacteroidetes | norank |
| OTU11754 | 110.92 | 97.50 | 74.17 | 94.19 | 36S | 36S | Proteobacteria | Steroidobacter |
| OTU9172 | 95.17 | 89.75 | 78.00 | 87.64 | 36S | 36S | Nitrospirae | norank |
| OTU587 | 89.08 | 52.50 | 61.67 | 67.75 | 36S | 36S | Bacteroidetes | Niastella |
| OTU8916 | 46.33 | 25.25 | 8.92 | 26.83 | 36S | 36S | Bacteroidetes | Sphingobacterium |
| OTU11317 | 38.25 | 13.08 | 13.25 | 21.53 | 36S | 36S | Verrucomicrobia | norank |
| OTU9417 | 30.67 | 20.83 | 8.83 | 20.11 | 36S | 36S | Proteobacteria | norank |
| OTU6811 | 28.00 | 17.42 | 7.92 | 17.78 | 36S | 36S | Proteobacteria | norank |
| OTU12434 | 26.00 | 17.00 | 6.83 | 16.61 | 36S | 36S | Bacteroidetes | norank |
| OTU3898 | 18.83 | 16.08 | 10.75 | 15.22 | 36S | 36S | Actinobacteria | norank |
| OTU3598 | 16.42 | 9.33 | 8.75 | 11.50 | 36S | 36S | Acidobacteria | norank |
| OTU247 | 7.67 | 5.50 | 4.58 | 5.92 | 36S | 36S | Bacteroidetes | norank |
| OTU1740 | 7.83 | 5.50 | 3.58 | 5.64 | 36S | 36S | Planctomycetes | norank |
| OTU8967 | 5.92 | 5.25 | 4.83 | 5.33 | 36S | 36S | Proteobacteria | norank |
| OTU7852 | 7.42 | 5.33 | 2.33 | 5.03 | 36S | 36S | Planctomycetes | Pirellula |
| OTU4808 | 6.67 | 4.25 | 4.00 | 4.97 | 36S | 36S | Chloroflexi | norank |
| OTU12055 | 8.08 | 5.42 | 1.08 | 4.86 | 36S | 36S | Bacteroidetes | norank |
| OTU6026 | 5.25 | 3.67 | 4.08 | 4.33 | 36S | 36S | Planctomycetes | norank |
| OTU7530 | 6.17 | 3.67 | 1.17 | 3.67 | 36S | 36S | Cyanobacteria | norank |
| OTU11872 | 6.25 | 1.67 | 2.67 | 3.53 | 36S | 36S | Proteobacteria | norank |
| OTU9509 | 4.42 | 2.17 | 3.83 | 3.47 | 36S | 36S | FBP | norank |
| OTU6213 | 3.83 | 2.83 | 2.67 | 3.11 | 36S | 36S | Planctomycetes | norank |
| OTU7975 | 4.92 | 3.42 | 1.00 | 3.11 | 36S | 36S | Bacteroidetes | norank |

Table S3. The relative abundance of the core microbiome for two soybean genotypes

| Genotype | OTU ID | Phylum | Class | Order | Family | Genus | Species | Relative abundance (%) |
| --- | --- | --- | --- | --- | --- | --- | --- | --- |
| T-genotype | OTU3795 | Proteobacteria | Gammaproteobacteria | Xanthomonadales | Xanthomonadaceae | Thermomonas | norank | 1.02 |
|  | OTU6911 | Proteobacteria | Alphaproteobacteria | Sphingomonadales | Sphingomonadaceae | norank | norank | 2.84 |
|  | OTU8585 | Proteobacteria | Alphaproteobacteria | Sphingomonadales | Sphingomonadaceae | norank | norank | 1.06 |
|  | OTU9044 | Proteobacteria | Alphaproteobacteria | Sphingomonadales | Erythrobacteraceae | norank | norank | 0.96 |
|  | OTU9116 | Proteobacteria | Betaproteobacteria | Methylophilales | Methylophilaceae | norank | norank | 0.81 |
|  | OTU9219 | Proteobacteria | Alphaproteobacteria | Rhizobiales | Rhizobiaceae | Agrobacterium | norank | 1.07 |
|  | OTU4334 | Nitrospirae | Nitrospira | Nitrospirales | Nitrospiraceae | Nitrospira | norank | 0.92 |
|  | OTU6274 | Bacteroidetes | Saprospirae | Saprospirales | Chitinophagaceae | norank | norank | 0.84 |
|  | OTU6533 | Bacteroidetes | Saprospirae | Saprospirales | Chitinophagaceae | Flavisolibacter | norank | 1.07 |
|  | OTU10447 | Actinobacteria | Actinobacteria | Actinomycetales | Micrococcaceae | Renibacterium | norank | 2.41 |
|  | OTU4280 | Acidobacteria | Acidobacteria-6 | iii1-15 | norank | norank | norank | 0.91 |
|  | OTU4827 | Acidobacteria | Chloracidobacteria | RB41 | Ellin6075 | norank | norank | 1.14 |
|  | OTU8697 | Acidobacteria | Chloracidobacteria | RB41 | norank | norank | norank | 2.09 |
|  | OTU10068 | Acidobacteria | Acidobacteria-6 | iii1-15 | norank | norank | norank | 1.12 |
| S-genotype | OTU3795 | Proteobacteria | Gammaproteobacteria | Xanthomonadales | Xanthomonadaceae | Thermomonas | norank | 1.06 |
|  | OTU6911 | Proteobacteria | Alphaproteobacteria | Sphingomonadales | Sphingomonadaceae | norank | norank | 2.76 |
|  | OTU7122 | Proteobacteria | Gammaproteobacteria | Pseudomonadales | Pseudomonadaceae | Pseudomonas | norank | 3.13 |
|  | OTU8585 | Proteobacteria | Alphaproteobacteria | Sphingomonadales | Sphingomonadaceae | norank | norank | 0.96 |
|  | OTU9044 | Proteobacteria | Alphaproteobacteria | Sphingomonadales | Erythrobacteraceae | norank | norank | 0.88 |
|  | OTU9116 | Proteobacteria | Betaproteobacteria | Methylophilales | Methylophilaceae | norank | norank | 0.90 |
|  | OTU9219 | Proteobacteria | Alphaproteobacteria | Rhizobiales | Rhizobiaceae | Agrobacterium | norank | 1.81 |
|  | OTU4334 | Nitrospirae | Nitrospira | Nitrospirales | Nitrospiraceae | Nitrospira | norank | 0.66 |
|  | OTU6274 | Bacteroidetes | Saprospirae | Saprospirales | Chitinophagaceae | norank | norank | 0.74 |
|  | OTU6533 | Bacteroidetes | Saprospirae | Saprospirales | Chitinophagaceae | Flavisolibacter | norank | 0.77 |
|  | OTU10447 | Actinobacteria | Actinobacteria | Actinomycetales | Micrococcaceae | Renibacterium | norank | 3.85 |
|  | OTU4280 | Acidobacteria | Acidobacteria-6 | iii1-15 | norank | norank | norank | 0.77 |
|  | OTU4827 | Acidobacteria | Chloracidobacteria | RB41 | Ellin6075 | norank | norank | 1.23 |
|  | OTU8697 | Acidobacteria | Chloracidobacteria | RB41 | norank | norank | norank | 1.63 |
|  | OTU10068 | Acidobacteria | Acidobacteria-6 | iii1-15 | norank | norank | norank | 0.96 |

Table S4 Topological characteristics of rhizobacterial networks for different treatments.

| Network metrics | CKT | CKS | 7T | 7S | 36T | 36S |
| --- | --- | --- | --- | --- | --- | --- |
| Number of nodes | 85 | 85 | 84 | 86 | 94 | 100 |
| Number of edges | 235 | 415 | 262 | 147 | 281 | 367 |
| Number of positive correlations | 204 | 323 | 213 | 140 | 201 | 279 |
| Number of negative correlations | 31 | 92 | 49 | 7 | 80 | 88 |
| Average degree (avgK) | 5.529 | 9.765 | 6.238 | 3.419 | 5.979 | 7.34 |
| Average weighted degree | 5.545 | 7.974 | 5.313 | 3.534 | 3.058 | 5.173 |
| Network diameter | 12 | 8 | 8 | 12 | 9 | 12 |
| Graph density | 0.066 | 0.116 | 0.075 | 0.04 | 0.064 | 0.074 |
| Modularity (M) | 0.933 | 0.603 | 1.172 | 0.913 | 2.785 | 1.415 |
| Interconnecting piece | 21 | 22 | 22 | 37 | 29 | 19 |
| Average clustering coefficient (avgCC) | 0.695 | 0.755 | 0.686 | 0.569 | 0.753 | 0.725 |
| Average path length (APL) | 4.158 | 2.871 | 3.63 | 4.5 | 3.636 | 4.153 |

Table S5. keystone species observed in rhizosphere soils of different treatments.

|  | OTU ID | degree | closeness centrality | betweenness centrality | Phylum | Class | Order | Family | Genus | Species |
| --- | --- | --- | --- | --- | --- | --- | --- | --- | --- | --- |
| CKT | OTU11321 | 15 | 0.295454545 | 118.4136364 | Verrucomicrobia | Verrucomicrobiae | Verrucomicrobiales | Verrucomicrobiaceae | Luteolibacter | norank |
|  | OTU8346 | 11 | 0.320987654 | 355.6414502 | Acidobacteria | Acidobacteria-6 | iii1-15 | norank | norank | norank |
|  | OTU9044 | 10 | 0.317073171 | 224.9725108 | Proteobacteria | Alphaproteobacteria | Sphingomonadales | Erythrobacteraceae | norank | norank |
| CKS | OTU6533 | 20 | 0.518518519 | 557.4393565 | Bacteroidetes | Saprospirae | Saprospirales | Chitinophagaceae | Flavisolibacter | norank |
|  | OTU6911 | 30 | 0.513761468 | 85.16376274 | Proteobacteria | Alphaproteobacteria | Sphingomonadales | Sphingomonadaceae | norank | norank |
|  | OTU8750 | 32 | 0.523364486 | 137.7622872 | Actinobacteria | Thermoleophilia | Gaiellales | Gaiellaceae | norank | norank |
| 7T | OTU1003 | 18 | 0.347826087 | 113.8733904 | Bacteroidetes | Saprospirae | Saprospirales | Chitinophagaceae | norank | norank |
|  | OTU2516 | 13 | 0.337349398 | 123.7489171 | Verrucomicrobia | Pedosphaerae | Pedosphaerales | norank | norank | norank |
|  | OTU2709 | 11 | 0.337349398 | 362.9856724 | Bacteroidetes | Cytophagia | Cytophagales | Cytophagaceae | norank | norank |
| 7S | OTU4362 | 9 | 0.266666667 | 152 | Acidobacteria | Chloracidobacteria | RB41 | norank | norank | norank |
|  | OTU8697 | 6 | 0.285714286 | 126 | Acidobacteria | Chloracidobacteria | RB41 | norank | norank | norank |
|  | OTU3795 | 5 | 0.274509804 | 215 | Proteobacteria | Gammaproteobacteria | Xanthomonadales | Xanthomonadaceae | Thermomonas | norank |
| 36T | OTU881 | 15 | 0.409090909 | 523.9860143 | Proteobacteria | Alphaproteobacteria | Rhizobiales | Bradyrhizobiaceae | Balneimonas | norank |
|  | OTU2516 | 21 | 0.406015038 | 230.866487 | Verrucomicrobia | Pedosphaerae | Pedosphaerales | norank | norank | norank |
|  | OTU3461 | 17 | 0.385714286 | 269.2095884 | Acidobacteria | Chloracidobacteria | RB41 | Ellin6075 | norank | norank |
| 36S | OTU3416 | 19 | 0.309782609 | 268.7812325 | Acidobacteria | Chloracidobacteria | RB41 | Ellin6075 | norank | norank |
|  | OTU5467 | 19 | 0.313186813 | 222.1984827 | Proteobacteria | Betaproteobacteria | Ellin6067 | norank | norank | norank |
|  | OTU1578 | 11 | 0.323863636 | 349.3870915 | Bacteroidetes | Saprospirae | Saprospirales | Chitinophagaceae | norank | norank |
